# The miR-214-3p/*CTSD* Axis Regulates Lysosomal Homeostasis in Porcine Intestinal Epithelial Cells: A Preliminary Study

**DOI:** 10.3390/biology15090693

**Published:** 2026-04-28

**Authors:** Huixia Wang, Ruifeng Zhong, Wenli Li, Yijia Tao, Yali Li

**Affiliations:** Hunan Provincial Key Laboratory of Animal Intestinal Function and Regulation, Hunan Normal University, Changsha 410081, China; 202320142760@hunnu.edu.cn (H.W.); 202470143026@hunnu.edu.cn (R.Z.); liyouyou06@gmail.com (W.L.); 202420142848@hunnu.edu.cn (Y.T.)

**Keywords:** miR-214-3p, *CTSD*, lysosome, intestinal epithelial cells, piglet

## Abstract

Lysosomes are crucial for the function of fetal vacuolated enterocytes in neonatal piglets, yet how they are regulated by miRNAs remains poorly defined. Therefore, this study aimed to elucidate how miRNAs govern lysosomal homeostasis in the developing intestine. Using a neonatal piglet model of lysosomal dysfunction induced by imipramine (IMI), we identified ssc-miR-214-3p as a key down-regulated miRNA implicated in lysosomal pathways. In IPEC-J2 enterocytes, the miR-214-3p mimic ameliorated IMI cytotoxicity by restoring cell viability and migration while suppressing apoptosis. Further analysis revealed that miR-214-3p directly reversed the lysosomal defects triggered by IMI treatment. Specifically, it alleviated lysosomal alkalinization and markedly restored acid phosphatase (ACP) activity, indicating a recovery of the acidic hydrolytic environment. This restoration was also accompanied by the preservation of lysosomal membrane integrity and a consequent reduction in the nuclear translocation of transcription factor EB (TFEB). Furthermore, cathepsin D (*CTSD*) was validated as a direct target of miR-214-3p by luciferase assay, and its overexpression reversed the protective effects of the mimic on lysosomal acidification and lysosome-associated membrane protein 1 (LAMP1) levels. Collectively, our findings reveal a novel miR-214-3p/*CTSD* axis that regulates lysosomal homeostasis during neonatal intestinal maturation, providing a potential therapeutic target for porcine intestinal disorders.

## Simple Summary

This study identified a key mechanism for protecting intestinal cells in newborn piglets, which involves the critical intracellular structures known as lysosomes. In experiments where lysosomes were damaged, a small molecule called miR-214-3p restored cell viability and alleviated lysosomal dysfunction by preventing drug-induced alkalinization, restoring acid phosphatase activity, and enhancing lysosomal membrane integrity. This protective effect is achieved through the precise targeting of the cathepsin D (*CTSD*). We found that the miR-214-3p/*CTSD* axis is crucial for maintaining lysosomal homeostasis in porcine intestinal epithelial cells. This discovery provides a potential target for future therapies aimed at improving intestinal health in piglets.

## 1. Introduction

The Neonatal piglets possess a unique population of fetal-type vacuolated enterocytes in the small intestine during the suckling period, characterized by large cytoplasmic vacuoles with a well-developed endosomal–lysosomal system [1]. These specialized enterocytes are essential for the uptake of maternal immunoglobulins and the digestion and absorption of milk-derived nutrients. Within the first three weeks after birth, these fetal-type vacuolated enterocytes are gradually replaced by mature enterocytes, indicating a developmental transition from intracellular to extracellular digestion [2]. Aberrant function or replacement of fetal enterocytes is associated with neonatal intestinal disorders and increased mortality [3,4]. Our previous study demonstrated that lysosomal dysfunction leads to significant growth retardation and metabolic defects in neonatal piglets, highlighting the critical role of lysosomes in intestinal maturation and nutrient processing during early life [5].

MicroRNAs (miRNAs) are ~22-nt non-coding RNAs that typically bind to the 3′ untranslated region (3′ UTR) of target mRNAs to induce degradation or translational repression [6]. Recent studies have revealed a vital role for miRNAs in neuroprotection, anti-infective immunity and lysosomal storage disorders through their regulation of autophagy-lysosome flux, mitochondrial reactive oxygen species (ROS) production, and apoptosis [7,8,9]. Their interactions with lysosomal membrane proteins (LMPs) also position them as potential therapeutic targets in cancer [10]. In piglet intestinal health, miRNAs have emerged as key regulators of intestinal development and immune homeostasis. For instance, Zou et al. (2019) [11] demonstrated that miRNAs are involved in the renewal of the small intestinal epithelial crypt-villus axis and further identified that miR-100 promotes the differentiation and apoptosis of porcine intestinal epithelial cells, while inhibiting their proliferation and migration. Other miRNAs, such as miR-146a, miR-132, miR-221-5p, and milk-derived exosomal miR-22-3p, have been shown to promote intestinal cell proliferation and to alleviate inflammatory injury and barrier disruption by targeting distinct pro-inflammatory or stress-related signaling cascades [12,13,14,15]. Nevertheless, it remains unclear how miRNAs precisely modulate lysosomal function at the molecular level in enterocytes during neonatal development.

Therefore, to investigate this, we first established a lysosomal dysfunction model in neonatal piglets using the lysosomal inhibitor imipramine (IMI) and profiled the resultant miRNA expression landscape by transcriptome sequencing. We then conducted in vitro functional assays to elucidate how key candidate miRNAs regulate lysosomal homeostasis and verify their molecular targets. This work aims to clarify the molecular mechanisms of miRNA-mediated lysosomal homeostasis in the developing piglet intestine, paving the way for novel interventions to improve neonatal gut health.

## 2. Materials and Methods

### 2.1. Animal Experimental Design

To minimize genetic variability, a major confounding factor in in vivo studies, six newborn female piglets (Duroc × (Landrace × Large Yorkshire)) with similar birth weights were selected from the same litter. Using a split-litter design, they were randomly assigned to two treatment groups (*n* = 3 per group). Piglets in the treatment group received a daily oral gavage of imipramine (IMI; Sigma-Aldrich, St. Louis, MO, USA) at a dose of 25 mg/kg body weight, while those in the control group received an equivalent volume of PBS. The IMI dosage was selected based on our previous study [5]. During the experimental period, all piglets were kept with their sow and had free access to suckling, with water provided ad libitum. After 7 days of treatment, all piglets were euthanized. Ileal tissue samples were collected, snap-frozen in liquid nitrogen, and stored at −80 °C for subsequent miRNA sequencing analysis.

### 2.2. miRNAs Identification and Pathway Enrichment Analysis

Small RNA was isolated from ileum tissues of neonatal piglets using TRIzol reagent (Life Technologies, New York, NY, USA) according to the manufacturer’s protocol. RNA sequencing was performed on the NovaSeq X Plus platform (Majorbio, Shanghai, China). Following quality control using Fastx-Toolkit (Version 0.0.14), clean reads were aligned to the swine reference genome (Sscrofa11.1) using Bowtie software (Version 1.2.3). Known miRNAs were annotated by comparing mapped reads to the miRBase database. Novel miRNAs were predicted based on the hairpin structures of miRNA precursors using miRDeep2 software (Version 2.0.1.3). The counts of known and predicted miRNAs in each sample are provided in Table S1. Differentially expressed miRNAs were identified using a threshold of |log2FC| > 0 and adjusted *p*-value ≤ 0.05 and were selected for further analysis. Target genes of both known and novel miRNAs were predicted using miRanda (version 2021.1.4) and RNAhybrid software (version 2.1.2) with default parameters. Functional enrichment of the target genes was performed through Gene Ontology (GO) and Kyoto Encyclopedia of Genes and Genomes (KEGG) pathway analyses. GO enrichment analysis was conducted by Goatools (version 1.4.5), with significance set at Bonferroni-corrected *p* < 0.05. KEGG pathway analysis was carried out using KOBAS 2.0 software, with an adjusted *p*-value cut-off of 0.05.

### 2.3. Cell Culture and miRNA Transfection of IPEC-J2 Cells

Porcine intestinal epithelial cells (IPEC-J2) were kindly provided by Dr. Yulong Yin (Institute of Subtropical Agriculture, Chinese Academy of Sciences) and have been maintained in our laboratory. Passages 70–90 were used for this study. Cells were cultured in DMEM/F12 medium (Gibco, Grand Island, NY, USA) supplemented with 10% heat-inactivated fetal bovine serum (FBS, Gibco), 100 U/mL penicillin (Invitrogen, Carlsbad, CA, USA) and 100 μg/mL streptomycin (Invitrogen), at 37 °C in a humidified incubator with 5% CO_2_. For transfection, IPEC-J2 cells were seeded into 24-well plates and grown to 60–80% confluence. miRNA mimics, inhibitors, and their corresponding negative controls (NC) (Ribio, Guangzhou, China) were diluted in Opti-MEM reduced-serum medium (Gibco) and mixed with Lipofectamine 2000 reagent (Invitrogen) according to the manufacturer’s instructions. The mixture was incubated at room temperature (RT) for 20 min to allow complex formation. The complexes were then added to the cells to achieve final concentrations of 50 nM for mimics and 100 nM for inhibitors. After 6 h of incubation, the transfection mixture was removed and replaced with fresh complete DMEM/F12 medium. Cells were cultured for an additional 24 h before further analysis. All transfections were performed in triplicate and repeated independently three times.

### 2.4. Quantitative Real-Time PCR (qRT-PCR) Analysis

Total RNA was extracted using TRIzol reagent (Life Technologies) according to the manufacturer’s instructions. RNA purity and concentration were determined on a Nanodrop 2000 spectrophotometer (Thermo Scientific, Waltham, MA, USA). To assess miR-214-3p expression, small RNA was reverse transcribed with the miRNA 1st-Strand cDNA Synthesis Kit (Accurate Biotechnology, Changsha, China), which included specific reverse transcription primers and U6 snRNA primers as internal controls. Forward primer sequence for miR-214-3p: 5′-CTGCGGCATCCACGAAACT-3′. For the detection of genes related to apoptosis, endocytosis and mitophagy, cDNA was synthesized using PrimeScript RT Master Mix (Takara, Dalian, China). qRT-PCR was then carried out as previously described [5], and relative gene expression levels were calculated using the 2^−ΔΔCT^ method. All primer sequences were designed with NCBI Primer-BLAST tool (https://www.ncbi.nlm.nih.gov/tools/primer-blast/index.cgi?LINK_LOC=BlastHome, accessed on 15 November 2023) and are listed in Table 1.

### 2.5. Cell Viability Assay

IPEC-J2 cells were seeded at 5 × 10^3^ cells/well in a 96-well plate and allowed to attach overnight. After 24 h of IMI exposure, 10 µL of CCK-8 reagent (Biosharp, Suzhou, China) was added to each well, and the plate was incubated for 4 h. Absorbance at 450 nm was read on a microplate reader (Biotek, Rochester, NY, USA). Each group had 4–6 replicates, and the experiment was independently repeated at least three times. Cell viability was calculated based on the absorbance values.

### 2.6. Cell Migration Assay

For cell migration assay, cells (untransfected, or transfected with miR-214-3p mimic or inhibitor) were cultured to full confluence in 6-well plates. After a 24 h treatment with or without IMI (75 µM), straight scratches were made through the center of each well, ensuring consistent width across all samples. The culture medium was aspirated, and the cell monolayer was gently washed 2–3 times with PBS to remove detached cells. Serum-free medium was then added to minimize cell proliferation during the assay. Images of the same field were recorded at 0, 6, 12, and 24 h under a microscope (Leica, Wetzlar, Germany). The scratched areas were quantified using ImageJ software (version 1.54a). Cell migration area (%) = [(Initial scratched area − Scratched area at final time point)/Initial scratched area] × 100.

### 2.7. Apoptosis Assay

Apoptosis was quantified with an Annexin V-Allophycocyanin/Propidium Iodide (AV-APC/PI) apoptosis detection kit (KeyGen, Nanjing, China). Briefly, adherent cells were detached with EDTA-free trypsin, washed twice with PBS by centrifugation (2000 rpm, 5 min, 4 °C), and then resuspended in 1× Binding Buffer. Subsequently, Annexin V-APC and PI were added to the cell suspension, followed by incubation for 10 min at RT in the dark. Finally, the samples were analyzed on a CytoFLEX flow cytometer (Beckman Coulter, Brea, CA, USA), and the data were analyzed using FlowJo software (v10, Tree Star, Ashland, OR, USA).

### 2.8. Measurement of Lysosomal pH with LysoSensor Probe

Lysosomal pH was measured using the ratiometric probe LysoSensor Yellow/Blue DND-160 (Thermo Fisher Scientific), following exposure to IMI (75 µM) or Bafilomycin A1 (Baf-A1, 100 nM) for 12 h. IPEC-J2 cells grown in clear-bottom black 96-well plates were rinsed and loaded with 2 µM probe in basic medium for 5 min at 37 °C. After PBS washes, fluorescence was measured using a microplate reader (Biotek) with dual-emission detection at 440 nm and 540 nm upon excitation at 360 nm. The 440 nm/540 nm ratio was used to calculate relative pH changes.

### 2.9. Lysosomal Acid Phosphatase Activity Assay

Acid phosphatase (ACP) activity in lysosomes was determined using a commercial kit (Acid Phosphatase Assay Kit; Beyotime, Shanghai, China). Following the manufacturer’s instructions, cells were lysed and centrifuged at 10,000 rpm for 5 min to obtain the supernatant. The supernatant was then incubated with the chromogenic substrate at 37 °C for 10 min. After adding 160 μL of stop solution to terminate the reaction, the absorbance was measured at 405 nm using a microplate reader (Biotek).

### 2.10. Lysosomal Membrane Permeability Assay

Lysosomal membrane integrity was evaluated using the Acridine Orange (AO) Staining Kit (Solarbio, Beijing, China) following the manufacturer’s instructions. Briefly, harvested cells were washed and resuspended in 1× Buffer, and the cell concentration was adjusted to 1 × 10^6^ cells/mL. A working staining solution was prepared by mixing the cell suspension with AO stain at a 19:1 ratio, followed by incubation at RT for 15 min in the dark. Subsequently, a drop of the stained suspension was mounted on a glass slide and immediately visualized under a fluorescence microscope (Leica, Wetzlar, Germany). The samples were excited at 488 nm, and emissions were collected using filters at 515 nm (for green) and 650 nm (for red).

### 2.11. Immunofluorescence

IPEC-J2 cells (untransfected, or transfected with miR-214-3p mimic or inhibitor) grown on glass coverslips were treated with 75 μM IMI for either 12 h (for LAMP1 detection) or 1 h (for TFEB detection). Following treatment, cells were fixed with 4% paraformaldehyde for 15 min at RT, permeabilized with 0.1% Triton X-100 (20 min, RT) and subsequently blocked with 1% BSA (30 min, RT). Coverslips were then incubated overnight at 4 °C with rabbit anti-LAMP1 (1:300; BIOSS, Beijing, China) or anti-TFEB (1:300; Proteintech, Rosemont, IL, USA), followed by CoraLite488-goat anti-rabbit secondary antibody (1:800; Proteintech) for 1 h at 37 °C. Nuclei were counterstained with DAPI (Abcam, Cambridge, UK). Slides were mounted in anti-fade medium and imaged on an Axio Imager 2 microscope (ZEISS, Oberkochen, Germany).

### 2.12. Dual-Luciferase Reporter Assay

DNA fragments containing the predicted miR-214-3p binding site within the 3′ UTR of CTSD (wild-type, WT) or corresponding mutants were cloned into the pmirGLO luciferase reporter vector (Sangon Biotech, Shanghai, China). IPEC-J2 cells were co-transfected with these constructed vectors together with either miR-214-3p mimics or mimic-NC (RiboBio, Guangzhou, China) using Lipofectamine 2000 reagent (Invitrogen). Luciferase activity was measured 48 h post-transfection using the Dual-Luciferase^®^ Reporter Assay System (Promega, Madison, WI, USA) according to the manufacturer’s protocol. Firefly luciferase activity was normalized to Renilla luciferase activity, and the results are presented relative to the control group.

### 2.13. CTSD Overexpression Assay

The cDNA sequence of porcine *CTSD* was cloned into the pcDNA3.1(+) vector (Sangon Biotech) to generate the *CTSD* overexpression plasmid (pcDNA-*CTSD*), with the empty pcDNA3.1(+) vector served as a negative control. IPEC-J2 cells were seeded in 12-well plates at a density of 1 × 10^5^ cells per well and cultured to 60–80% confluence. Transfections were performed with 1 µg plasmid, 50 nM miR-214-3p mimic or mimic-NC (Ribobio, Guangzhou, China) using 2 µL Lipofectamine 2000 (Invitrogen) per well according to the manufacturer’s protocol. After 6 h of transfection, cells were treated with 75 μM IMI for 12 h before subsequent analysis.

### 2.14. Statistical Analysis

Data are presented as mean ± SEM. Statistical significance was determined using an unpaired Student’s *t* test with GraphPad Prism version 7.0 (San Diego, CA, USA). A *p* value < 0.05 was considered statistically significant.

## 3. Results

### 3.1. miR-214-3p Was Down-Regulated and Enriched in Lysosomal-Related Pathways in IMI-Treated Piglets

**Figure 1:**
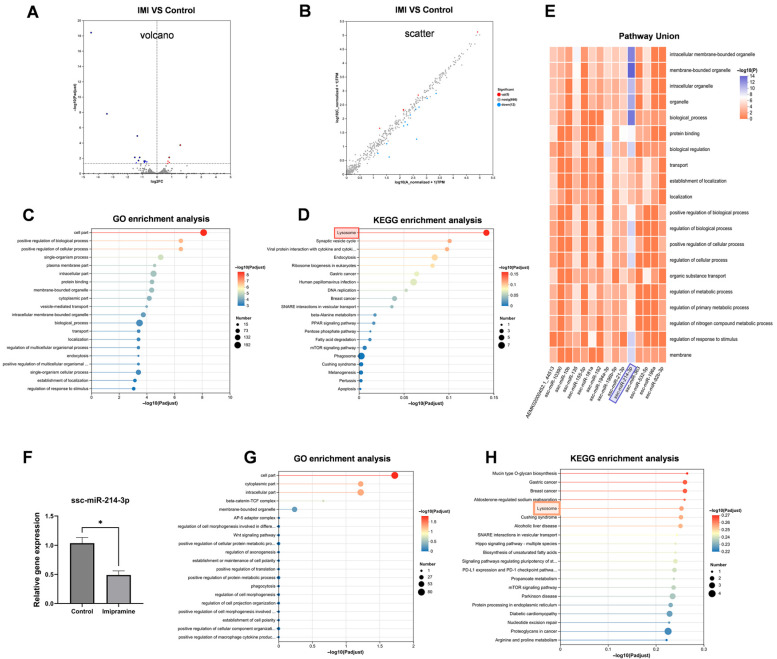
Identification of miR-214-3p as a key regulator in piglets following imipramine (IMI) treatment. (**A**) Volcano plot and (**B**) scatter plot of differentially expressed miRNAs. Red dots indicate upregulated miRNAs; blue dots indicate downregulated miRNAs; gray dots indicate non-significantly expressed miRNAs. Each dot represents an individual miRNA. (**C**) GO and (**D**) KEGG enrichment of the union of predicted targets of all differentially expressed miRNAs. (**E**) Pathway union analysis of potential target genes. (**F**) qRT-PCR validation of miR-214-3p expression (mean ± SEM; *n* = 3; * *p* < 0.05). (**G**) GO and (**H**) KEGG enrichment analysis of potential target genes of miR-214-3p. The lysosome pathway is highlighted with a rectangular box for emphasis.

As illustrated in the volcano and scatter plots, a total of 17 differentially expressed miRNAs were identified in piglets following IMI treatment, including 5 up-regulated and 12 down-regulated miRNAs (Figure 1A,B). GO and KEGG enrichment analysis of the combined predicted targets of all differential miRNAs revealed significant enrichment in terms related to cell part and lysosome (Figure 1C,D). Pathway-union analysis further indicated that miR-214-3p exhibited the strongest enrichment in organelle-related pathways (Figure 1E). The down-regulated expression of miR-214-3p in IMI-treated piglets was further confirmed by qRT-PCR (Figure 1F). Accordingly, GO and KEGG analysis of miR-214-3p targets also showed predominant enrichment in cell part and lysosome-related categories (Figure 1G,H).

### 3.2. Protective Role of miR-214-3p Against IMI-Induced Cytotoxicity in IPEC-J2 Cells

**Figure 2:**
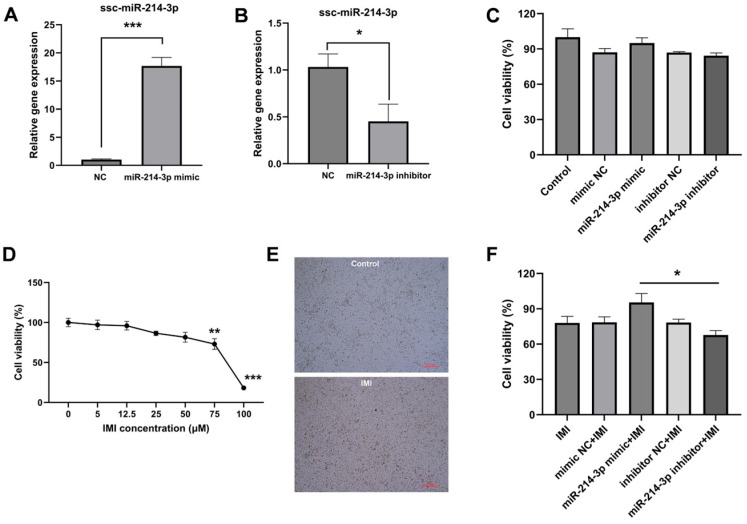
Effects of miR-214-3p transfection and IMI treatment on IPEC-J2 cell viability. (**A**,**B**) qRT-PCR verification of miR-214-3p over-expression (mimic) and knock-down (inhibitor). (**C**) Cell viability after miR-214-3p transfection. (**D**) Cell viability after 24 h exposure to IMI treatment. (**E**) Representative cell images after IMI treatment (scale bar = 200 µm). (**F**) Cell viability of miR-214-3p-transfected IPEC-J2 cells after treatment with IMI (75 µM, 24 h). Data = mean ± SEM; *n* = 4–6; * *p* < 0.05; ** *p* < 0.01; *** *p* < 0.001.

**Figure 3:**
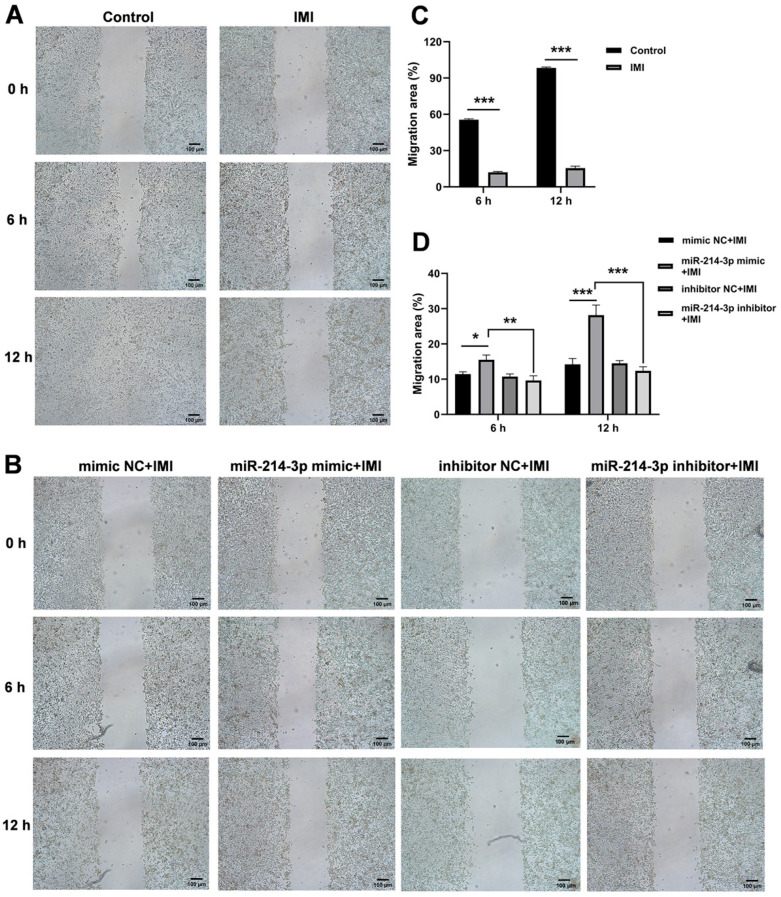
Effects of miR-214-3p on IPEC-J2 cell migration. (**A**,**B**) Representative images of cell migration at 0, 6, and 12 h post-wounding. Images are representative of three independent experiments; Scale bar = 100 µm. (**C**,**D**) Quantification of migration area. Data = mean ± SEM; *n* = 15; * *p* < 0.05, ** *p* < 0.01, *** *p* < 0.001 versus respective control groups.

**Figure 4:**
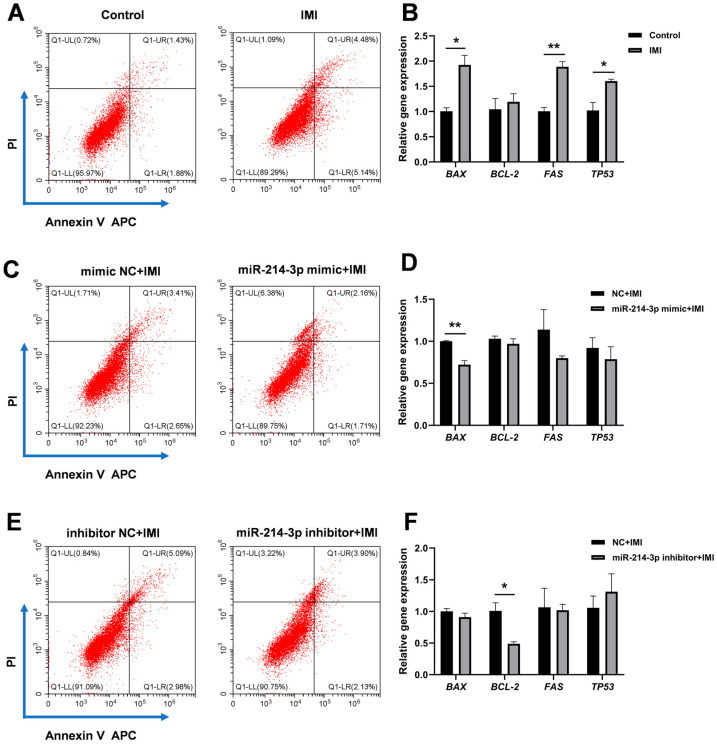
Effects of miR-214-3p on IMI-induced apoptosis in IPEC-J2 cells. (**A**) Representative AV-APC/PI flow cytometry plots and (**B**) relative expression levels of apoptosis-related genes in non-transfected IPEC-J2 cells after IMI treatment (75 µM, 24 h). (**C**) Flow cytometry analysis and (**D**) corresponding qRT-PCR data in cells pre-transfected with miR-214-3p mimic followed by IMI treatment. (**E**) Flow cytometry analysis and (**F**) qRT-PCR results in cells pre-transfected with miR-214-3p inhibitor followed by IMI treatment. Flow cytometry plots: The lower-left quadrant (AV-APC^−^/PI^−^) represents viable cells; the lower-right quadrant (AV-APC^+^/PI^−^) represents early apoptotic cells; the upper-right quadrant (AV-APC^+^/PI^+^) represents late apoptotic or necrotic cells; the upper-left quadrant (AV-APC^−^/PI^+^) represents cells with primary necrosis or mechanical membrane damage. Numbers indicate the percentage of cells in each quadrant. Data are representative of three independent experiments; * *p* < 0.05, ** *p* < 0.01 versus respective control groups.

As shown in Figure 2A,B, transfection with miR-214-3p mimic successfully elevated intracellular miR-214-3p levels, whereas the inhibitor reduced them, confirming the efficiency of the transfection model. Neither the mimic nor the inhibitor affected basal cell viability (Figure 2C). IMI (75 μM, 24 h) markedly reduced viability (Figure 2D,E), and this impairment was relieved by pre-transfection with the miR-214-3p mimic (Figure 2F). Moreover, IMI treatment significantly inhibited IPEC-J2 cell migration at both 6 h and 12 h after wounding (Figure 3A,C). Notably, this suppression was mitigated by pre-transfection with the miR-214-3p mimic, but not by its inhibitor (Figure 3A,C). Furthermore, IMI induced clear apoptosis in IPEC-J2 cells, evidenced by an increased ratio of AV-APC/PI-positive cells and up-regulation of the pro-apoptotic genes Bcl-2-associated X protein (*BAX*), Fas cell surface death receptor (*FAS*), and tumor protein p53 (*TP53*) (Figure 4A,B). Pre-transfection with the miR-214-3p mimic attenuated this effect, reducing both the AV/PI staining ratio and *BAX* expression level (Figure 4C,D). Conversely, the inhibitor further suppressed the expression of the anti-apoptotic gene *BCL-2* (Figure 4F).

### 3.3. miR-214-3p Does Not Affect IMI-Induced Endocytosis and Mitophagy

**Figure 5:**
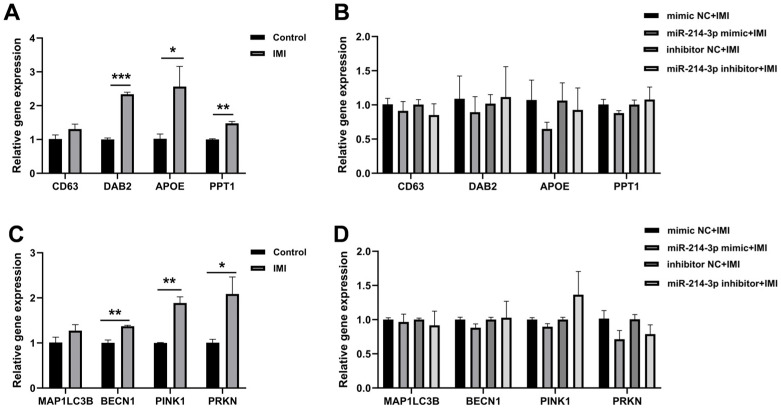
Effects of miR-214-3p on endocytosis- and mitophagy-related gene expression. (**A**) Expression levels of endocytosis-related genes in non-transfected IPEC-J2 cells exposed to IMI (75 µM, 24 h). (**B**) Expression levels of endocytosis-related genes in miR-214-3p mimic- or inhibitor-transfected cells after IMI treatment. (**C**) Expression levels of mitophagy-related genes in non-transfected IPEC-J2 cells treated with IMI. (**D**) Expression levels of mitophagy-related genes in miR-214-3p mimic- or inhibitor-transfected cells after IMI treatment. Data are representative of three independent experiments; * *p* < 0.05, ** *p* < 0.01, *** *p* < 0.001 versus respective control groups.

Exposure to IMI significantly up-regulated the endocytosis-related genes disabled homolog 2 (*DAB2*), apolipoprotein E (*APOE*) and palmitoyl-protein thioesterase 1 (*PPT1*) (Figure 5A) and the mitophagy genes beclin 1 (*BECN1*), PTEN-induced putative kinase 1 (*PINK1*) and parkin RBR E3 ubiquitin-protein ligase (*PRKN*) (Figure 5C). However, neither the miR-214-3p mimic nor its inhibitor altered these IMI-induced mRNA profiles (Figure 5B,D). This finding suggests that the protective effects of miR-214-3p are not mediated through the regulation of endocytosis or mitophagy pathways.

### 3.4. miR-214-3p Modulates IMI-Induced Lysosomal Alkalinization and Impairment of Acid Phosphatase Activity

**Figure 6:**
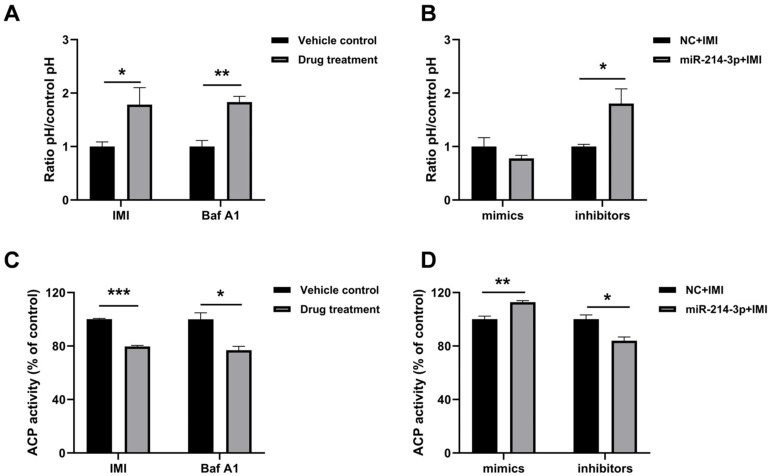
Impact of miR-214-3p on lysosomal pH and acid phosphatase (ACP) activity in IPEC-J2 cells. (**A**) Lysosomal pH following IMI or Bafilomycin A1 (Baf A1) exposure. Cells were treated with IMI (75 µM) or Baf A1 (100 nM) for 12 h. The vehicle controls were PBS for IMI and dimethyl sulfoxide (DMSO) for Baf A1. pH was determined using the fluorescent probe LysoSensor Yellow/Blue DND-160. (**B**) Lysosomal pH after IMI treatment in cells pre-transfected with miR-214-3p mimic or inhibitor. (**C**) ACP activity following IMI or Baf A1 exposure. (**D**) ACP activity after IMI treatment in cells pre-transfected with miR-214-3p mimic or inhibitor. Data are representative of three independent experiments; * *p* < 0.05, ** *p* < 0.01, *** *p* < 0.001 versus respective control groups.

As shown in Figure 6A, IMI treatment significantly increased lysosomal pH, an effect comparable to that induced by the positive control Baf A1 (a well-known lysosomal inhibitor). Notably, pre-transfection with the miR-214-3p inhibitor further enhanced this alkalinizing effect (Figure 6B). Moreover, both IMI and Baf A1 treatment led to a decrease in ACP activity (Figure 6C). Pre-transfection with the miR-214-3p mimic attenuated the IMI-induced reduction in ACP activity, whereas the inhibitor exacerbated it (Figure 6D).

### 3.5. miR-214-3p Enhances Lysosomal Membrane Integrity upon IMI Challenge

**Figure 7:**
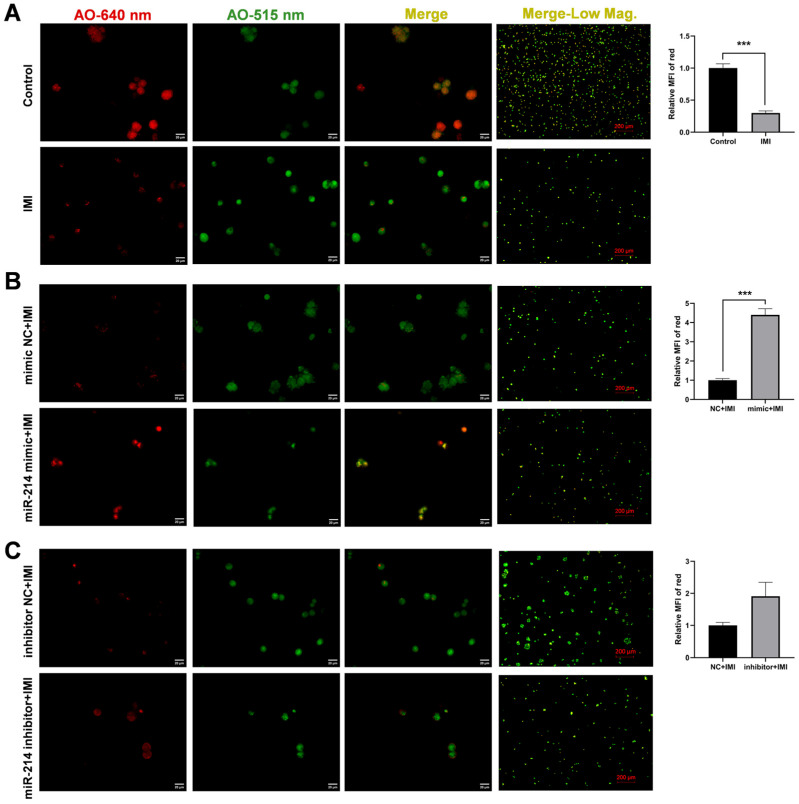
Effects of miR-214-3p on lysosomal membrane permeability. Lysosomal membrane permeability in (**A**) non-transfected IPEC-J2 cells, (**B**) miR-214-3p mimic-transfected cells, and (**C**) miR-214-3p inhibitor-transfected cells following IMI exposure (75 µM, 12 h). Scale bars: 20 µm for 400× and 200 µm for 50× magnifications (Low Mag.). Fluorescence channels: red (640 nm, protonated AO in acidic lysosomes) and green (515 nm, free AO and AO-DNA complexes in nuclei/cytosol). The mean fluorescence intensity (MFI) of the red fluorescence signal was quantified. Data are representative of three independent experiments; *** *p* < 0.001 versus respective control groups.

Lysosomal membrane permeability was assessed using AO staining. As shown in Figure 7A, IMI evoked a significant decrease in red MFI following IMI exposure, indicating enhanced lysosomal membrane permeability. This effect was partially restored by pre-transfection with the miR-214-3p mimic, which increased the relative red MFI compared to the NC group after IMI treatment (Figure 7B). In contrast, the inhibitor had no significant effect on IMI-induced lysosomal membrane permeabilization (Figure 7C).

### 3.6. miR-214-3p Inhibits IMI-Induced Nuclear Translocation of TFEB

**Figure 8:**
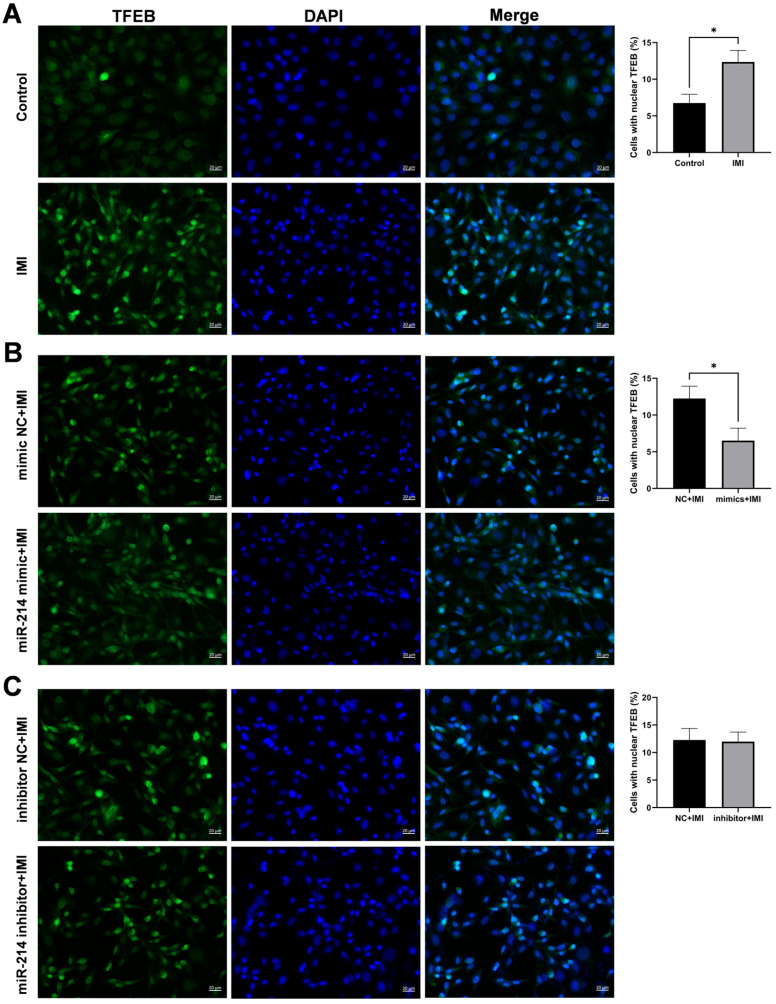
Effects of miR-214-3p on TFEB nuclear translocation. TFEB (green) nuclear translocation in (**A**) non-transfected, (**B**) miR-214-3p mimic-transfected, and (**C**) miR-214-3p inhibitor-transfected cells following IMI exposure (magnification 400×). Images are representative of three independent experiments; Scale bar = 20 µm. * *p* < 0.05.

As shown in Figure 8, IMI treatment triggered nuclear translocation of TFEB, the principal transcription factor governing lysosomal biogenesis (Figure 8A). The nuclear translocation was, however, partially blocked by pre-transfection with the miR-214-3p mimic, which significantly reduced translocation compared to the NC group (Figure 8B). In contrast, the miR-214-3p inhibitor did not significantly affect IMI-induced TFEB nuclear translocation (*p* > 0.05) (Figure 8C).

### 3.7. Validation of CTSD as a Direct Target Gene of miR-214-3p

**Figure 9:**
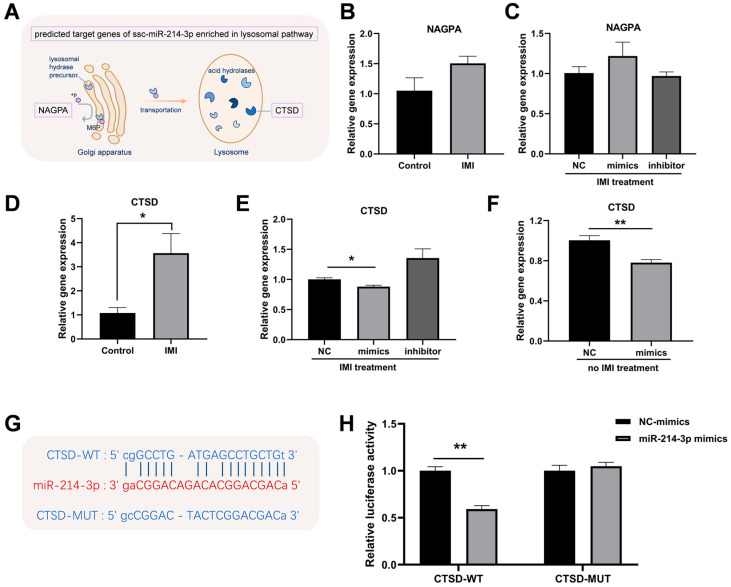
Validation of *CTSD* as a direct target gene of miR-214-3p. (**A**) Predicted targets of miR-214-3p. Relative *NAGPA* mRNA expression in (**B**) non-transfected IPEC-J2 cells and (**C**) miR-214-3p mimic- or inhibitor-transfected cells after IMI (IMI) exposure. Relative *CTSD* mRNA expression in (**D**) non-transfected and (**E**) miR-214-3p mimic- or inhibitor-transfected cells after IMI exposure. (**F**) Relative *CTSD* mRNA expression in miR-214-3p mimic-transfected cells without IMI treatment. (**G**) Predicted miR-214-3p binding site within the *CTSD* 3′ UTR. (**H**) Luciferase activity of reporter plasmids containing wild type (WT) or mutant (MUT) *CTSD* binding sites. Data are representative of three independent experiments; * *p* < 0.05, ** *p* < 0.01 versus respective control groups.

As shown in Figure 9A, both N-acetylglucosamine-1-phosphodiester alpha-N-acetylglucosaminidase (*NAGPA*) and cathepsin D (*CTSD*) were predicted as potential targets of miR-214-3p using bioinformatic tools. However, qRT-PCR showed that IMI treatment did not alter *NAGPA* mRNA expression in either non-transfected or miR-214-3p-transfected cells (Figure 9B,C). Conversely, *CTSD* was up-regulated by IMI alone (Figure 9D) but was significantly down-regulated when the miR-214-3p mimic was present, irrespective of IMI treatment (Figure 9E,F). The predicted binding site of miR-214-3p within the *CTSD* 3′ UTR was illustrated in Figure 9G. Luciferase activity was significantly reduced in cells co-transfected with the miR-214-3p mimic and the WT reporter plasmid, but remained unchanged in those transfected with the MUT construct (Figure 9H).

### 3.8. Overexpression of CTSD Reverses the Protective Effects Induced by miR-214-3p

**Figure 10:**
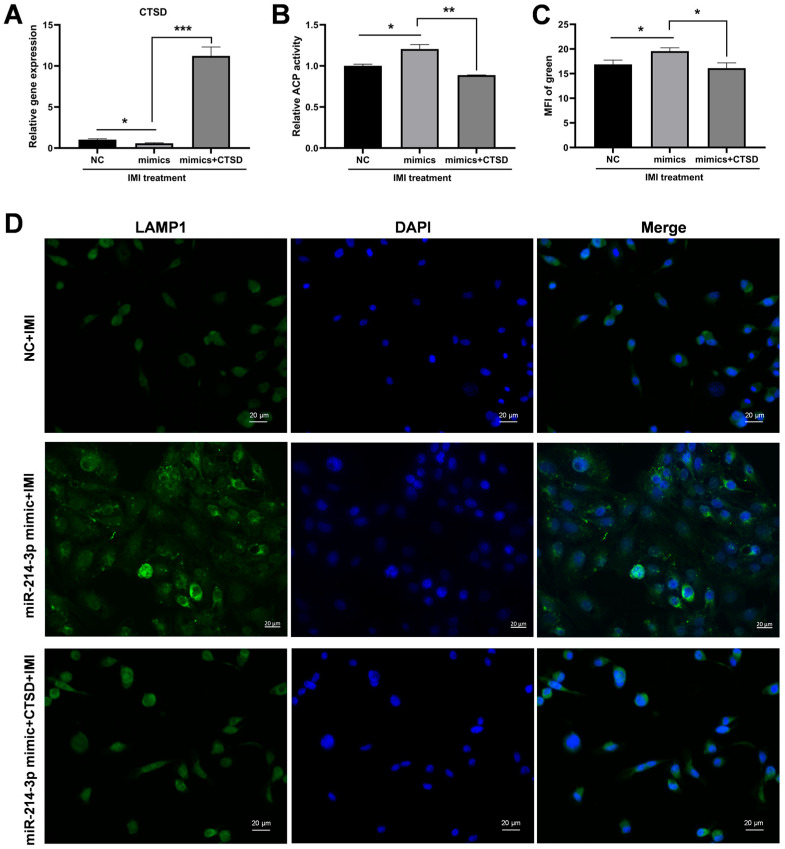
*CTSD* overexpression counteracts the beneficial effects of miR-214-3p. (**A**) Relative *CTSD* expression in cells co-transfected with miR-214-2p and *CTSD* plasmid after IMI exposure. (**B**) ACP activity in cells co-transfected with miR-214-2p and *CTSD* plasmid following IMI exposure. (**C**) MFI of LAMP1 in cells co-transfected with miR-214-2p and *CTSD* plasmid post IMI exposure. (**D**) Representative LAMP1 staining images in cells co-transfected with miR-214-2p and *CTSD* plasmid after IMI treatment (magnification 400×). Images are representative of three independent experiments. Scale bar = 20 µm. The mean fluorescence intensity (MFI) of the green fluorescence signal was quantified; * *p* < 0.05, ** *p* < 0.01, *** *p* < 0.001 versus respective control groups. Green: LAMP1 (lysosome marker).

As shown in Figure 10A, under IMI treatment, *CTSD* mRNA expression was significantly downregulated in miR-214-3p mimic-transfected cells compared to the NC group but was restored upon co-transfection with the *CTSD* plasmid. Consistently, after IMI exposure, the increase in ACP activity observed in mimic-transfected cells was abolished by *CTSD* overexpression (Figure 10B). Furthermore, expression of LAMP1, a lysosomal membrane marker, was markedly reduced in cells co-transfected with the miR-214-3p mimic and *CTSD* plasmid following IMI exposure, compared to the mimic-only controls (Figure 10C,D).

## 4. Discussion

In our previous study, we demonstrated that IMI, a cationic amphiphilic drug, triggers lysosomal dysfunction in immature fetal-type enterocytes and contributes to growth retardation in neonatal piglets [5]. To further explore the underlying mechanism, we investigated the changes in miRNA expression profiles and identified miR-214-3p as a significantly down-regulated miRNA in the ileal tissue of IMI-treated piglets. Pathway analysis revealed that its predicted targets were predominantly enriched in organelle-related pathways, particularly the lysosomal pathway.

Accumulating evidence has implicated miR-214-3p in various key cellular processes, including cell proliferation, migration, apoptosis, and metastasis in specific cancer types [16,17,18,19]. In inflamed chondrocytes, miR-214-3p exerted its protective anti-inflammatory effects by down-regulating the NF-κB signaling pathway [20]. Moreover, miR-214-3p has been reported to inhibit macrophage ferroptosis, suppress M1 polarization, and attenuate the ensuing inflammatory response [21]. These findings, together with our own data, prompted us to further investigate its function in the IPEC-J2 cell model. Gain-of-function and loss-of-function experiments clearly demonstrated that miR-214-3p effectively alleviated IMI-induced cytotoxicity, as evidenced by the restoration of cell viability and migration, and the suppression of apoptosis. Specifically, following IMI treatment, the miR-214-3p mimic increased cell viability compared to the inhibitor group, while also significantly enhancing the migration area relative to the NC group. Moreover, miR-214-3p mimic reduced the expression of the pro-apoptotic gene *BAX*, while its inhibitor further suppressed the anti-apoptotic gene *BCL-2*, indicating a pivotal role for miR-214-3p in regulating IMI-induced apoptotic cell death. The cytoprotective role of miR-214-3p, as observed in this study, is supported by prior findings in diverse cellular models. In line with our observation, Cheng et al. (2005) [16] demonstrated that miR-214 decreases apoptosis in HeLa cells. Similarly, Yang et al. (2013) [22] reported that miR-214 knockdown inhibits gastric cancer cell proliferation, migration, and invasion via the PTEN pathway. These consistent results across different models point to a conserved role for miR-214-3p in promoting cell survival.

Pathway analysis, together with our previous finding, prompted us to examine lysosomal function and define the downstream pathways mediating the protective effects of miR-214-3p. Lysosomes are acidic, membrane-bound organelles that contain approximately 70 acid hydrolases, including ACP, and thus serve as the primary hub of intracellular degradation [23]. Consequently, their dysfunction may lead to severe cellular impairment and even cell death [24]. In the current study, IMI treatment caused classic lysosomal dysfunction in IPEC-J2 cells, such as lysosomal alkalinization and reduced ACP activity, effects comparable to those of Baf A1 (another well-established lysosomal inhibitor). These results are consistent with Albright et al. (2023) [25], who showed that IMI alters lysosomal pH and thereby inhibits multiple pH-sensitive enzymes in macrophage lysosomes. Moreover, IMI evoked marked lysosomal leakage in IPEC-J2 cells by increasing membrane permeabilization, a key hallmark of lysosomal injury [26]. Transfection with the miR-214-3p mimic significantly restored ACP activity and enhanced lysosomal membrane stability. Conversely, the miR-214-3p inhibitor aggravated the impairment, leading to further lysosomal alkalinization and a further decrease in ACP activity. Notably, the protective effect of miR-214-3p was lysosome-specific, as it did not affect the IMI-induced upregulation of endocytosis and mitophagy genes. These results strongly demonstrate that miR-214-3p mediates cellular protection by maintaining lysosomal homeostasis, thereby ensuring the functional integrity of this central degradative organelle, which is essential for cell survival. Our findings align with a previous study by Lou et al. (2022) [24], which demonstrated that miR-504-5p overexpression stabilizes the lysosomal membrane, inhibits necroptosis, and maintains cell viability after ischemic injury. In addition to pH and ACP activity, future studies measuring lysosomal degradation capacity, another key indicator of lysosomal homeostasis, would further support the role of miR-214-3p in regulating lysosomal function.

The transcription factor EB (TFEB) has emerged as a master regulator of autophagy-lysosomal biogenesis by promoting the coordinated lysosomal expression and regulation (CLEAR) network. This transcription factor is activated by diverse cellular stresses, including nutrient deficiency, hypoxia, lysosomal stress, and mitochondrial damage [27,28]. In our study, IMI treatment triggered significant nuclear translocation of TFEB, an effect that was attenuated when cells were transfected with the miR-214-3p mimic prior to IMI exposure, suggesting a protective role of miR-214-3p in maintaining lysosomal function. Several miRNAs can directly target the TFEB transcript or indirectly regulate components of its post-translational modification machinery, thereby shaping its downstream transcriptional programs [27]. According to Guo et al. (2021) [29], miR-30b-5p can bind to CLEAR elements in the nucleus, thereby inhibiting TFEB-dependent transcription and consequently regulating lysosomal biogenesis and autophagy. However, the precise mechanistic interplay between miR-214-3p and TFEB remains unclear. Further studies are needed to determine whether their interaction is direct or indirect, and to define the functional consequences of this crosstalk for lysosomal homeostasis, particularly in the context of IMI-induced lysosomal stress.

Cathepsin D (CTSD) is an aspartic protease responsible for protein turnover within lysosomes. CTSD displays dual, location-dependent functions: the secreted pro-form promotes cell proliferation, whereas the intracellular mature form triggers apoptosis [30]. Given its critical role in cell signaling, dysregulated CTSD has been implicated in numerous pathological conditions [31]. Kågedal et al. (2001) [32] reported that CTSD translocation from lysosome to cytosol is an early event in naphthazarin-induced fibroblast apoptosis, positioning CTSD upstream of the caspase cascade. Moreover, in hyperglycemia-induced cardiomyocyte injury, high glucose was shown to increase *CTSD* expression, induce lysosomal membrane permeabilization, and trigger CTSD release, thereby contributing to cell damage [33]. In contrast, another study has demonstrated that *CTSD* expression can be effectively downregulated by miR-326 at both the mRNA and protein levels [34]. In this study, IMI treatment significantly upregulated *CTSD* expression, an effect largely reversed by the miR-214-3p mimic. Subsequently, a luciferase reporter assay confirmed a direct targeting relationship between miR-214-3p and *CTSD*. In functional experiments, overexpressing *CTSD* directly counteracted the increase in ACP activity induced by the miR-214-3p mimic upon IMI treatment. Moreover, under the same conditions, the concurrent reduction in LAMP1, a structural marker of lysosomal membrane integrity, further indicates a broader lysosomal impairment. These findings suggest that aberrantly elevated CTSD induced by IMI disrupts lysosomal function, and that miR-214-3p may exert its protective effects, at least in part, by downregulating *CTSD*. Our findings align with the mechanism established by Yan et al. (2022) [35], in which miR-214-3p attenuates LPS-induced myocardial injury by targeting cathepsin B (*CTSB*), leading to reduced oxidative stress and apoptosis. However, another study reported that miR-352 obstructs autophagy process via targeting *LAMP2* and cathepsin L1 (*CTSL1*), contributing to the pathogenesis of acute pancreatitis [36]. Future studies involving *CTSD* knockdown are warranted to confirm its role as the primary functional mediator of miR-214-3p, whereas the investigation of additional lysosome-related targets will help to fully elucidate this regulatory network.

## 5. Conclusions

Collectively, the current study, by integrating in vivo and in vitro experiments, elucidates the critical role of ssc-miR-214-3p in IMI-induced intestinal toxicity and its underlying molecular mechanism. Our primary finding is that miR-214-3p exerts its cytoprotective effects against IMI exposure by directly targeting *CTSD* and thereby modulating lysosomal function, suggesting that the miR-214-3p-*CTSD*-lysosome axis could serve as a novel therapeutic target for developing innovative health management strategies in neonatal piglets. Future studies incorporating additional lysosomal stressors and larger in vivo cohorts are warranted to validate the broader relevance of the miR-214-3p/*CTSD* axis in lysosomal homeostasis and to confirm whether direct modulation of miR-214-3p restores lysosomal function and improves intestinal physiology in vivo.

## Figures and Tables

**Table 1:** Primers used for qRT-PCR analysis.

Genes	Primers	Sequences (5′-3′)
*BAX*	Forward	CCGATCTCGAAGGAAGTCCA
	Reverse	TTGAGAATTGCACACCAACC
*BCL* *-2*	Forward	ATCCCAGCCTCCGTTATCCT
	Reverse	GCTGACGGCAACTTCAACTG
*FAS*	Forward	TTGAGAATTGCACACCAACC
	Reverse	CGATACCATTCTTCCGAACG
*TP53*	Forward	GTCGGCTCTGACTGTACCAC
	Reverse	TTCAGCTCCAAGGCGTCATT
*MAP1LC3B*	Forward	CCGAACCTTCGAACAGAGAG
	Reverse	AGGCTTGGTTAGCATTGAGC
*BECN1*	Forward	AGGAGCTGCCGTTGTACTGT
	Reverse	CACTGCCTCCTGTGTCTTCA
*PINK1*	Forward	CTCTGGTCGACTACCCCGAT
	Reverse	ATGACGAGGAAGAGTGTCCG
*PRKN*	Forward	CCAAACCGGATGAGTGGTGA
	Reverse	CTTGTCAGAGGTCGGGTGTG
*CD63*	Forward	ATGGCGGTGGAAGGAGGA
	Reverse	CTACATCACCTCGTAGCCACTTCG
*DAB2*	Forward	ATCAGACATCTTTGCTCCTCC
	Reverse	CTAAAGACGCTGGGTTCCAT
*APOE*	Forward	GGTGCAGTCCCTGTCTGA
	Reverse	CTCTATCAGCTCCGTCAGTTC
*PPT1*	Forward	GGCTCAGAGATGCCCCATAC
	Reverse	CGGGGTCCACAATGGAATCA
*NAGPA*	Forward	AAGTGAAGCAGTGTCTCCCG
	Reverse	CAGAAGGAAAACCAGGGCCA
*CTSD*	Forward	CAGGGCGAGTACATGATCCC
	Reverse	CGACACCTTGAGCGTGTAGT
*β-actin*	Forward	CTGCGGCATCCACGAAACT
	Reverse	AGGGCCGTGATCTCCTTCTG

## Data Availability

All data will be available upon reasonable request from the corresponding author.

## Supplementary Materials

The following supporting information can be downloaded at: https://www.mdpi.com/article/10.3390/biology15090693/s1, Table S1: Counts of known and predicted miRNAs in each sample.

## Abbreviations

The following abbreviations are used in this manuscript: 3′ UTR = 3′ untranslated region; ACP = acid phosphatase; AO = acridine orange; AV-APC/PI = Annexin V-Allophycocyanin/Propidium Iodide; Baf A1 = Bafilomycin A1; BAX = Bcl-2-associated X protein; CLEAR = coordinated lysosomal expression and regulation; CTSB = cathepsin B; CTSD = cathepsin D; CTSL1 = cathepsin L1; DMSO = dimethyl sulfoxide; FAS = Fas cell surface death receptor; GO = Gene Ontology; IMI = imipramine; KEGG = Kyoto Encyclopedia of Genes and Genomes; LAMP1 = lysosome-associated membrane protein 1; LMP = lysosomal membrane protein; MFI = mean fluorescence intensity; miRNA = microRNA; MUT = mutant; NAGPA = N-acetylglucosamine-1-phosphodiester alpha-N-acetylglucosaminidase; NC = negative control; TP53 = tumor protein p53; ROS = reactive oxygen species; RT = room temperature; TFEB = transcription factor EB; WT = wild type.
