# Supplementary material for: The miR-214-3p/CTSD Axis Regulates Lysosomal Homeostasis in Porcine Intestinal Epithelial Cells: A Preliminary Study
Source: Biology (Basel). 2026 Apr 28;15(9):693. doi: 10.3390/biology15090693 (PMC13162880; doi:10.3390/biology15090693)
Supplement: Supplementary file 1 [file biology-15-00693-s001.zip › biology-4266859-supplementary.pdf]

Table S1. Counts of known and predicted miRNAs in each sample.

Sample	known miRNAs	novel miRNAs	total
Control1	359	187	546
Control2	353	152	505
Control3	364	220	584
Imipramine1	358	194	552
Imipramine2	357	194	551
Imipramine3	358	193	551
